# A Case of Right‐Sided Heart Failure and Severe Pulmonary Hypertension Secondary to Partial Anomalous Pulmonary Venous Connection

**DOI:** 10.1002/rcr2.70438

**Published:** 2025-12-09

**Authors:** Ahmed Tareq Alabsi, Khalifa Abdulrahman Yusuf, Alya Salman Aldoseri, Shadi Fayez Kanhosh, Abdulrahman Hasan Al‐Madani, Ahmed Taha Shaarawy

**Affiliations:** ^1^ Department of Emergency Medicine Bahrain Defence Force Hospital‐Royal Medical Services Riffa Bahrain; ^2^ Department of Internal Medicine Bahrain Defence Force Hospital‐Royal Medical Services Riffa Bahrain

**Keywords:** partial anomalous pulmonary venous connection, pulmonary hypertension, right‐sided heart failure and vasoreactivity testing

## Abstract

Partial anomalous pulmonary venous connection (PAPVC) is a rare congenital anomaly in which one or more pulmonary veins drain into the systemic venous circulation. PAPVC is often misdiagnosed as idiopathic pulmonary hypertension in the adult population. We report a case of a 48‐year‐old woman with long‐standing pulmonary hypertension who presented with recurrent pulmonary oedema and right‐sided heart failure. Transthoracic echocardiography showed severe pulmonary hypertension with preserved left ventricular function. Computed tomographic pulmonary angiography excluded pulmonary embolism but confirmed an anomalous drainage of the right upper pulmonary vein into the superior vena cava, consistent with isolated PAPVC without an atrial septal defect. Right heart catheterization confirmed severe precapillary pulmonary hypertension with a positive vasoreactivity response to adenosine. She was successfully managed medically with diltiazem and diuretics, showing sustained clinical improvement. This case highlights isolated PAPVC as a critical, under‐recognised cause of severe pulmonary hypertension in adults.

## Graphical abstract

We present a case of isolated partial anomalous pulmonary venous connection (PAPVC) in a 48‐year‐old woman with severe pulmonary hypertension and right‐sided heart failure. CT pulmonary angiography revealed anomalous drainage of the right upper pulmonary vein into the superior vena cava, with no associated atrial septal defect. This case underscores the importance of considering PAPVC as a rare but treatable cause of pulmonary hypertension in adults.

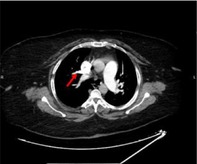

## 1 Introduction

Partial anomalous pulmonary venous connection (PAPVC) is a rare form of congenital heart defect in which one or more branches, but not all, of the pulmonary veins drain either directly to the right atrium or through the systemic veins [1]. These patients have a left‐to‐right shunt, resulting in decreased blood flow in the systemic veins. This physiologic defect is similar to that of atrial septal defect (ASD) [1]. If the shunt is significant, it can lead to right‐sided heart failure, pulmonary hypertension, and in advanced stages, these patients may be at increased risk for the development of Eisenmenger's complex. The prevalence of this condition, as determined by autopsies, ranges from 0.4% to 0.7% [2], with most cases associated with an ASD. Isolated PAPVC without interatrial communication is rare and may remain undiagnosed until adulthood. Recognition is critical, as timely identification can prevent progression to right‐sided dysfunction. We report a case of PAPVC where the pulmonary vein drains into the superior vena cava in the absence of an atrial septal defect, leading to significant and severe pulmonary hypertension.

## 2 Case Report

**FIGURE 1:**
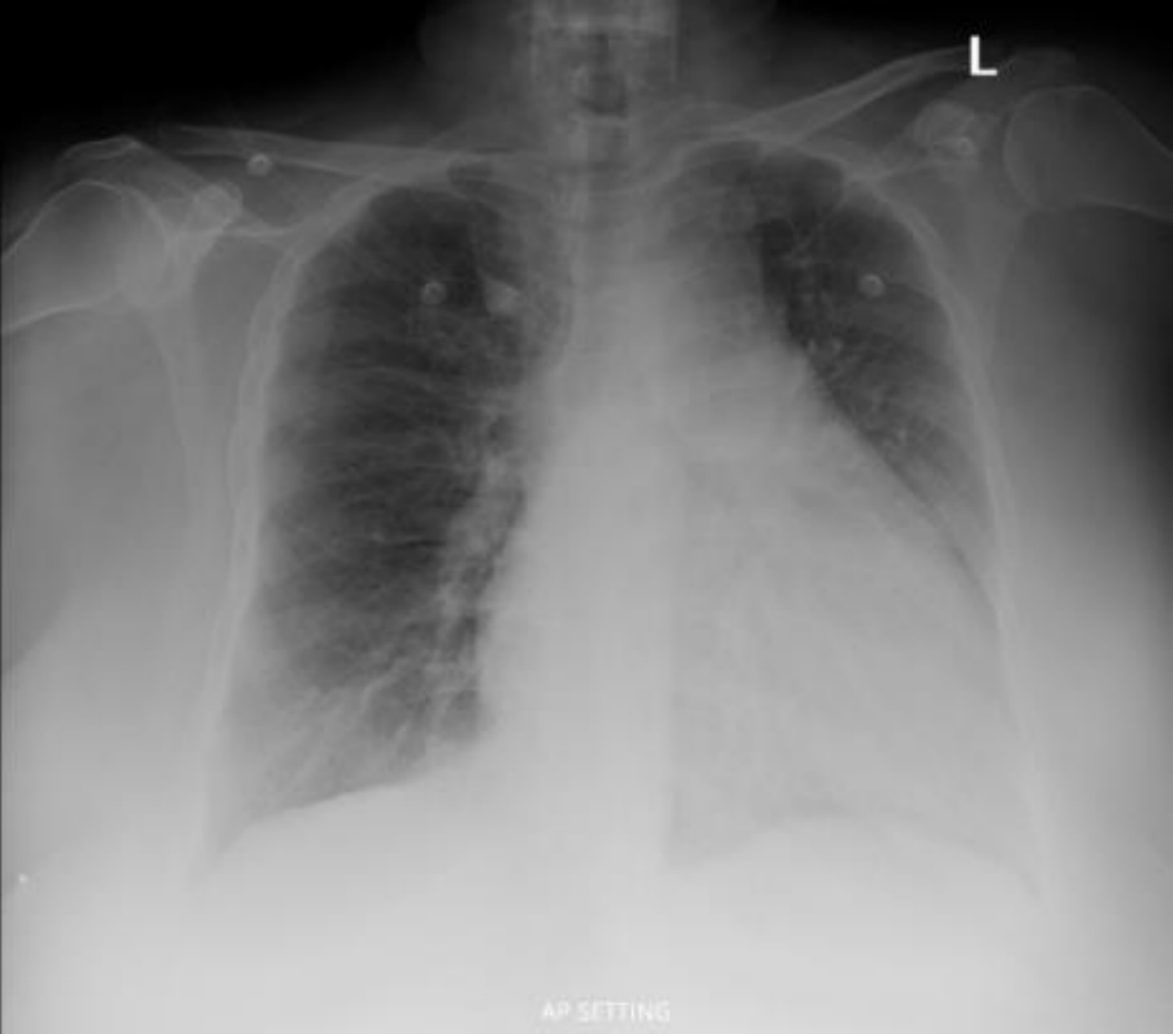
Anteroposterior chest x‐ray showing cardiomegaly with prominent right‐sided chambers and pulmonary congestion consistent with right‐sided heart failure.

A 48‐year‐old woman with type 2 diabetes, hypothyroidism, gastroesophageal reflux disease and previously diagnosed pulmonary hypertension presented with 2 days of worsening dyspnea and cough. She was compliant with aspirin, bisoprolol, indapamide, furosemide and atorvastatin. On arrival, she was stable with an oxygen saturation of 92% on 4 L/min of oxygen. Clinical examination revealed a loud second heart sound and bibasal crackles. NT‐proBNP was 652 pg/mL. Chest radiography demonstrated prominent pulmonary vascular markings consistent with pulmonary plethora, rather than classical pulmonary oedema (Figure 1).

Following the diagnostic sequence recommended by the 7th World Symposium on Pulmonary Hypertension (WSPH), transthoracic echocardiography demonstrated preserved left ventricular systolic function (LVEF 60%), normal LV cavity size, right ventricular dilatation (RVD1 3.8 cm), and borderline RV systolic function (TAPSE 2.0 cm). Moderate tricuspid regurgitation was present. The estimated RV systolic pressure was 95 mmHg. An agitated saline study showed no evidence of an interatrial shunt.

**FIGURE 2:**
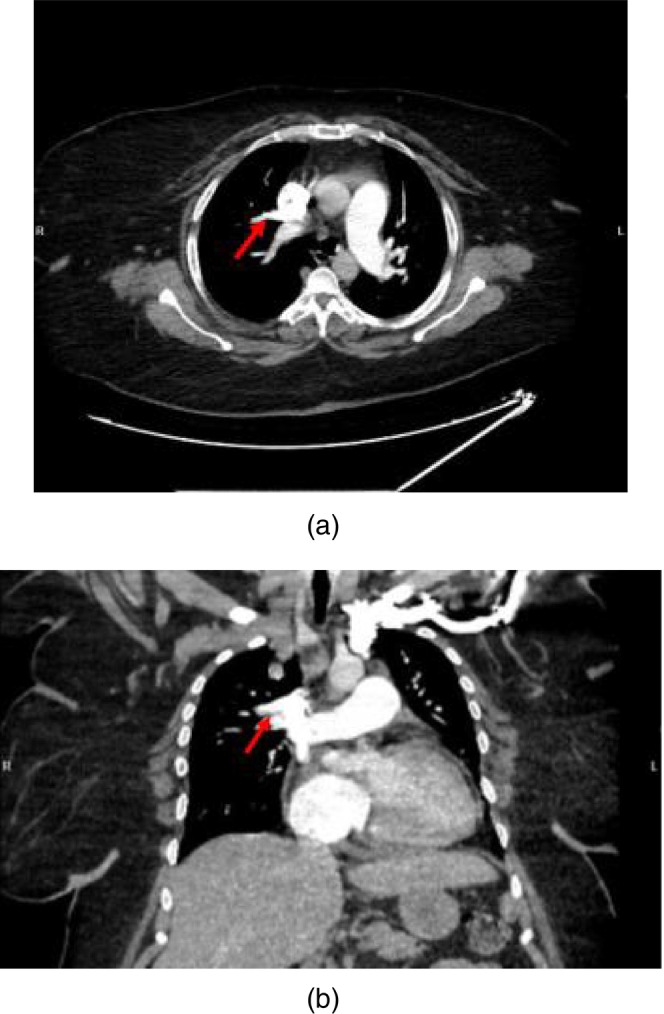
(a) Axial contrast‐enhanced CT chest demonstrating markedly dilated pulmonary artery and right heart chamber consistent with severe pulmonary hypertension. An anomalous vascular channel, seen coursing from the right upper lobe to the superior vena cava, represents partial anomalous pulmonary venous return. (b) Coronal contrast‐enhanced CT chest demonstrating markedly dilated pulmonary artery and enlarged right cardiac chambers consistent with severe pulmonary hypertension.

High‐resolution CT imaging demonstrated normal lung parenchyma, excluding intrinsic lung disease. CT pulmonary angiography confirmed the absence of pulmonary embolism and identified an isolated partial anomalous pulmonary venous connection (PAPVC), with anomalous drainage of the right upper pulmonary vein into the superior vena cava (Figure 2a,b).

She underwent invasive hemodynamic evaluation at a tertiary centre. Coronary angiography showed no significant disease. Right‐heart catheterization demonstrated the following pressures:

- Aortic: 160/76/102 mmHg
- RA: 16/15/16 mmHg
- RV: 73/31/28 mmHg
- PA: 73/46/58 mmHg
- PCWP: 21/26/17 mmHg
- LVEDP: 15 mmHg

Hemodynamic calculations showed:

- Cardiac output: 2.2 L/min (Fick)
- Cardiac index: 4.8 L/min/m^2^ (Fick)
- PVR: 8.0 Wood units
- SVR: 8.6 Wood units

Pulmonary artery oxygen saturation was 73% (aortic 88%), suggesting ongoing left‐to‐right shunting. Because only aortic and pulmonary artery saturations were measured, sequential chamber data were not available, and a formal step‐up assessment or Qp:Qs calculation could not be performed. Nonetheless, the disparity between the systemic arterial saturation (92%) and the pulmonary artery saturation (73%) is physiologically consistent with residual left‐to‐right flow in keeping with PAPVC, even without a calculated shunt ratio.

Vasoreactivity testing with incremental intravenous adenosine reduced mean PAP to 34–36 mmHg and increased cardiac output to 4.75 L/min. Although vasoreactivity testing is not routinely recommended for CHD‐associated pulmonary hypertension, it was performed due to diagnostic uncertainty and limited availability of selective pulmonary vasodilators. Given the elevated PVR and absence of a surgically correctable defect, she was managed medically with diltiazem 200 mg daily, the only locally available vasodilator option, alongside optimised diuretics. Although repeat invasive hemodynamic assessment was not performed, the patient demonstrated sustained clinical stability over the following 6 months, with improved exercise tolerance and no further episodes of decompensated right‐sided heart failure.

## 3 Discussion

Partial anomalous pulmonary venous connection (PAPVC) is often difficult to recognise, especially when it presents in adulthood with non‐specific respiratory or heart‐failure symptoms. The anomaly results from incomplete incorporation of the embryological pulmonary venous plexus into the left atrium, leading one or more pulmonary veins to drain into the systemic venous circulation [3]. Although PAPVC may be suspected on routine transthoracic echocardiography, definitive anatomical assessment commonly requires cardiac CT or MRI.

According to the 7th World Symposium on Pulmonary Hypertension (WSPH), this case is classified under Group 1.4.4 pulmonary arterial hypertension (PAH) associated with congenital heart disease, reflecting the combination of precapillary hemodynamics and isolated anomalous venous drainage without an interatrial communication. This classification is clinically relevant because diagnostic evaluation and treatment considerations differ from those for idiopathic or heritable PAH.

The physiological consequences of PAPVC follow a recognizable pattern. In early disease, anomalous drainage produces a left‐to‐right shunt with an increased Qp:Qs ratio, resulting in chronically elevated pulmonary blood flow. Persistent exposure to flow‐related shear stress promotes pulmonary vascular remodelling and rising pulmonary vascular resistance (PVR). As resistance increases, shunt magnitude decreases and Qp:Qs approaches 1, signalling later‐stage physiology. With further progression, bidirectional or right‐to‐left shunting may develop, eventually leading to Eisenmenger physiology. In this patient, the raised PVR together with an elevated pulmonary artery oxygen saturation was consistent with late left‐to‐right shunt physiology, although a formal Qp:Qs ratio could not be derived due to incomplete saturation profiling.

Vasoreactivity testing played a role in this case but must be interpreted with caution. Although well‐defined criteria exist for idiopathic, heritable and drug‐associated PAH, there are no accepted criteria for vasoreactivity testing in CHD‐associated PAH, and routine testing is not recommended in this subgroup. In this instance, testing was performed because of diagnostic uncertainty and limited access to selective pulmonary vasodilators, and the observed response supported the decision to trial calcium channel blocker therapy.

Management of PAPVC may be surgical or medical depending on symptoms, shunt magnitude and concurrent cardiac indications [4]. However, attempts to reduce PVR medically to permit later surgical repair have not proven successful and are not recommended in current practice. Pulmonary vasodilator therapy can be used in patients deemed high‐risk for surgery or as a bridge to surgery. In addition, pulmonary vasodilators may be used in patients with either isolated pre‐capillary pulmonary hypertension or post‐capillary hypertension, provided that the pre‐capillary component is defined as peripheral vascular resistance greater than 2, along with evidence of improvement in hemodynamic parameters [5]. With elevated PVR and no surgically correctable anatomy, our patient was appropriately managed medically and demonstrated sustained clinical improvement.

## Author Contributions

A.T.A., K.A.Y., A.S.A., S.F.K, A.H.A. and A.T.S. were directly involved in this patient's care. A.T.A., K.A.Y. and S.F.K. conceptualised the project. A.T.A. and K.A.Y. authored the manuscript. K.A.Y., A.S.A., S.F.K, A.H.A. and A.T.S. reviewed the manuscript.

## Consent

The authors declare that written informed consent was obtained for the publication of this manuscript and accompanying images using the consent form provided by the Journal.

## Conflicts of Interest

The authors declare no conflicts of interest.

## Data Availability

The data that support the findings of this study are available from the corresponding author upon reasonable request.
